# Magnetic resonance imaging in Alzheimer’s disease and mild cognitive impairment

**DOI:** 10.1007/s00415-018-9016-3

**Published:** 2018-08-17

**Authors:** Avinash Chandra, George Dervenoulas, Marios Politis, for the Alzheimer’s Disease Neuroimaging Initiative

**Affiliations:** Neurodegeneration Imaging Group (NIG), Maurice Wohl Clinical Neuroscience Institute, Institute of Psychiatry, Psychology and Neuroscience (IoPPN), King’s College London, 125 Coldharbour Lane, Camberwell, London, SE5 9NU UK

**Keywords:** Magnetic resonance imaging, Neuropathology, Alzheimer’s disease, Mild cognitive impairment

## Abstract

Research utilizing magnetic resonance imaging (MRI) has been crucial to the understanding of the neuropathological mechanisms behind and clinical identification of Alzheimer’s disease (AD) and mild cognitive impairment (MCI). MRI modalities show patterns of brain damage that discriminate AD from other brain illnesses and brain abnormalities that are associated with risk of conversion to AD from MCI and other behavioural outcomes. This review discusses the application of various MRI techniques to and their clinical usefulness in AD and MCI. MRI modalities covered include structural MRI, diffusion tensor imaging (DTI), arterial spin labelling (ASL), magnetic resonance spectroscopy (MRS), and functional MRI (fMRI). There is much evidence supporting the validity of MRI as a biomarker for these disorders; however, only traditional structural imaging is currently recommended for routine use in clinical settings. Future research is needed to warrant the inclusion for more advanced MRI methodology in forthcoming revisions to diagnostic criteria for AD and MCI.

## Introduction

Alzheimer’s disease (AD) is a neurodegenerative disorder and the most common cause of dementia. Mild cognitive impairment (MCI) is the prodromal form of AD and is characterized by neurocognitive dysfunction, but not to the extent of dementia, and minor difficulties in functional ability. The neuropathological hallmarks of AD include neurofibrillary tangles (NFTs) and beta-amyloid (Aβ) neuritic plaques. The AD brain contains increased levels of hyperphosphorylated tau. In this state, the main functions of normal tau are disrupted and the polymerization of paired helical filaments or NFTs, which are correlated with synaptic loss, occurs. Overproduction of amyloid precursor protein is also characteristic in AD, which results in elevated levels of Aβ_42_ and neuritic plaque formation. This exerts oxidative and inflammatory stress, which contributes to neuronal damage [1].

Through the in vivo visualization of neuropathology, magnetic resonance imaging (MRI) research has been paramount in the clinical identification of MCI and AD. Diagnostic criteria recommend the consideration of abnormalities on structural MRI [2, 3]. More advanced MR techniques include diffusion tensor imaging (DTI), arterial spin labelling (ASL), magnetic resonance spectroscopy (MRS), and functional magnetic resonance imaging (fMRI), which have not yet been established for routine clinical use. The aim of this review will be to provide an overview of the application of the various MR modalities in AD and MCI. Another clinically useful neuroimaging technology is positron emission tomography (PET) [4]; however, this is beyond the scope of the current work.

## Structural imaging

**Fig. 1:**
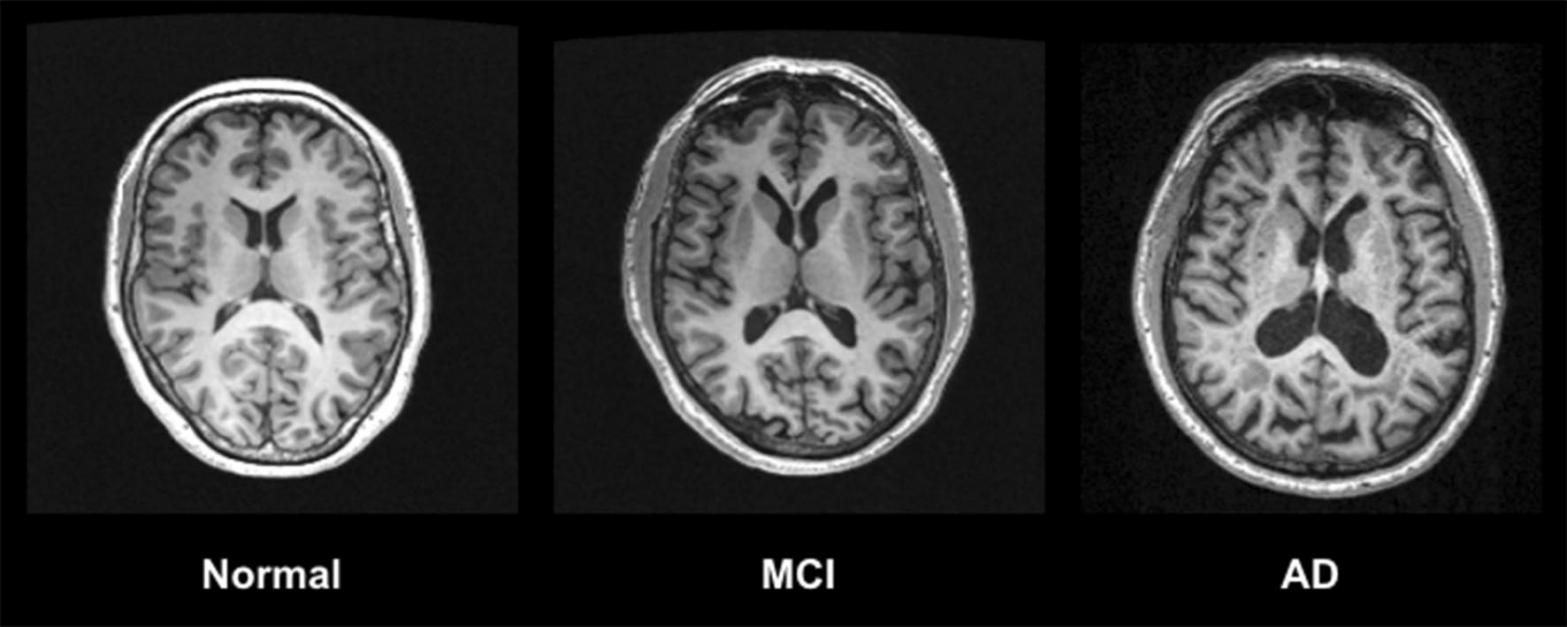
T1-weighted MRI imaging using an MPRAGE (Magnetisation Prepared Rapid Gradient Echo) sequence shows decreased GM volume in an AD patient compared to a healthy control and intermediate GM decline in a patient with MCI

Structural imaging modalities reveal brain atrophy and other static tissue abnormalities (Table 1; Fig. 1). Progression of atrophy follows Braak staging [5] and is first observed in medial temporal lobe (MTL) structures, including the entorhinal cortex (ERC) and hippocampus [6, 7]. Compared to controls, hippocampal volumes for AD patients are reduced by 26–27% and ERC volumes by 38–40% [6]. MCI patients show intermediate levels of MTL atrophy [7]. The presence of diffuse hippocampal atrophy is related to deficits in executive functioning and memory for AD patients [8]. As the disease progresses, atrophy advances to the remainder of the MTL where grey matter (GM) loss occurs in the medial temporal gyrus, parahippocampus, parahippocampal and fusiform gyri, and temporal pole [9]. Nesteruk and colleagues [10] found that MTL atrophy discriminates those who will convert from MCI to AD from non-converters. It also differentiates AD from dementia with Lewy bodies (DLB) and Parkinson’s disease with dementia (PDD), where AD patients show the greatest reductions in hippocampal volume [11, 12].

**Table 1:** Research studies examining region-specific patterns of neuropathology in AD and MCI using structural MRI

Study	Imaging modality	Sample	Main findings
Du et al. [6]	Structural MRI	20 AD, 25 cognitively normal (CN)	AD patients demonstrated GM loss in the hippocampus and ERC, with a higher atrophy rate in the ERC
Pennanen et al. [7]	Structural MRI	48 AD, 65 MCI, 59 controls	Hippocampal and ERC atrophies were found in AD and MCI patients, with MCI patients showing intermediate levels
Li et al. [9]	Structural MRI	64 AD, 72 controls (14 with AD on follow-up)	Early in the course of AD, the ERC and hippocampus are the primary sites of atrophy. In later stages, other MTL brain structures are affected
Cavedo et al. [13]	Structural MRI	19 AD, 19 controls	GM reductions were demonstrated in the amygdala for AD patients
Thomann et al. [14]	Structural MRI	21 early AD, 21 controls	Atrophy of the olfactory bulb tract was found for AD patients
Guo et al. [15]	Structural MRI	13 AD, 14 controls	GM reductions in parahippocampal gyrus, middle and superior temporal gyrus, insula, parietal lobule, thalamus, hippocampus, and cingulate gyrus were demonstrated for AD patients
De Jong et al. [16]	Structural MRI	69 probable AD, 70 subjects with memory complaints	Compared to subjects with memory complaints, GM loss was shown in the putamen and thalamus for AD patients
Kilimann et al. [19]	Structural MRI	134 AD, 41 MCI, 148 controls	Volumetric reductions in brain areas within the basal forebrain cholinergic system were displayed for AD and MCI patients
Duarte et al. [20]	Structural MRI	14 probable AD, 32 MCI, 14 controls	Frontal, parietal and temporal lobe atrophies were found for AD patients and frontal and temporal GM losses were present for MCI patients
Vasavada et al. [22]	Structural MRI	15 AD, 21 MCI, 27 CN	Brain atrophy was displayed in the hippocampus and the primary olfactory cortex for AD and MCI patients
Tabatabaei-Jafari et al. [23]	Structural MRI	191 AD, 398 MCI, 229 CN	GM reductions in the cerebellum were found for AD patients
Lee et al. [24]	Structural MRI	50 AD, 50 controls	Volumetric reductions in the brainstem were displayed in AD patients
Capizzano et al. [27]	Structural MRI	81 probable AD, 19 controls	A high degree of WMHs was found in AD patients: 70% in the frontal lobe, 22% in the parietal lobe, 3.5% in the temporal lobe, and 1% in the occipital lobe

Additional limbic structures including the amygdala, olfactory bulb tract, cingulate gyrus, and thalamus are impacted in AD [13–16]. GM loss in these regions is associated with cognitive dysfunction and neuropsychiatric symptomatology [17, 18]. As the disease progresses, atrophy spreads to cortical regions. Frontal, parietal, and temporal brain areas experience volumetric reductions, and so do the putamen and basal forebrain cholinergic system [15, 16, 19, 20]. Cholinergic abnormalities in AD have been further highlighted through the use of molecular imaging technologies [21]. Atrophy is also found in the primary olfactory cortex [22], in addition to lower-level brain areas including the cerebellum and brainstem [23, 24]. MCI is notable for frontal and temporal GM loss, and atrophy in the primary olfactory cortex and some basal forebrain cholinergic system structures [19, 20, 22]. No volumetric differences were found between AD patients with and without hypertension [25].

**Fig. 2:**
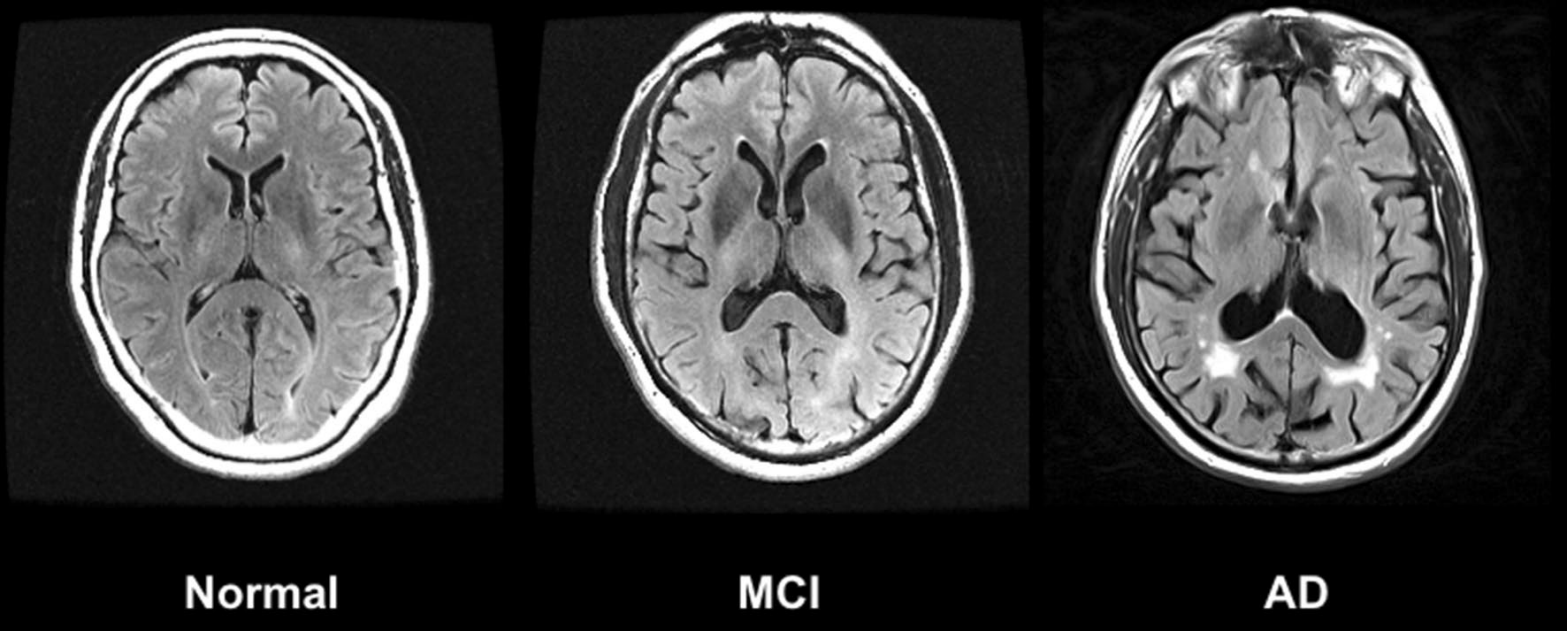
T2-weighted MRI imaging using a FLAIR (Fluid Attenuated Inversion Recovery) sequence shows increased WMHs in an AD patient compared to a healthy control and intermediate levels of WMHs in a patient with MCI

Structural MRI scans can also display white matter hyperintensities (WMHs), which indicate demyelination and axonal loss [26] (Table 1; Fig. 2). Compared to controls, patients with AD demonstrate greater WMHs with the majority in frontal lobe [27]. For patients along the AD spectrum, WMHs correlate with hippocampal atrophy [28], in addition to neuropsychological impairment and psychiatric disturbances [29, 30]. Considering differential diagnoses, patients with vascular dementia (VaD) have higher volumes of WMHs than in AD [31]. Periventricular WMHs are predictive of progression from MCI to AD, with an increase of one point in WMH rating associated with a 59% increased risk of phenoconversion [32].

## Advanced MR techniques

DTI utilizes the displacement of water molecules to measure white matter tract integrity (Table 2). The primary metrics of DTI include mean diffusivity (MD) or the average rate of water molecule diffusivity and fractional anisotropy (FA) or the variability associated with diffusion [33]. In AD, increased MD is noted in frontal, occipital, parietal, and temporal areas including the hippocampus; however, in MCI, these increases are absent in frontal and occipital regions. In AD, decreased FA is localized to the cingulum, corpus callosum, superior lateral fasciculus and uncinate fasciculus and throughout temporal, occipital and frontal white matter. Patients with MCI display a similar pattern, but with no FA irregularities in occipital and parietal areas [34]. MD increases in the basal forebrain are associated with increased risk of progression from MCI to AD [35], and FA and MD abnormalities are associated with memory and executive dysfunction [36, 37]. Diffusivity metrics also discriminate AD from other dementias where reduced FA is present in frontal areas for frontotemporal dementia (FTD) compared to AD, and increased MD is present in parietal and temporal regions for AD in contrast to DLB [38, 39]. However, DTI technology shows particular sensitivity to motion, which could lead to artifacts that might skew results. Comparatively long scanning times could increase the probability of such errors [40], indicating that this technique may not be particularly well suited for practical clinical use.

**Table 2:** Research studies examining region-specific patterns of neuropathology in AD and MCI using advanced MR modalities

Study	Imaging modality	Sample	Main findings
Sexton et al. [34]	DTI	Meta-analysis of 41 studies	MD increases were found globally in WM in AD and in temporal and parietal WM in MCI. FA decreases were found in temporal, occipital and frontal WM in AD and frontal and temporal WM in MCI
Alexopoulos et al. [41]	ASL	19 AD, 24 MCI, 24 controls	Hypoperfusion was noted in parietal, temporal, and occipital cortex, and the precuneus in MCI and AD patients
Mak et al. [42]	ASL	13 AD, 15 controls	Reductions in CBF were found in the hippocampus and posterior cingulate for patients with AD
Dai et al. [43]	ASL	37 AD, 29 MCI, 38 controls	In MCI, decreases in CBF were found in the posterior cingulate and precuneus and increases in CBF were found in the hippocampus, basal ganglia, and amygdala. In AD decreases in CBF were found in frontal, parietal, temporal, orbitofrontal cortex, and the precuneus and increases in CBF were found in the anterior cingulate gyrus. Compared to MCI patients, AD patients showed decreased CBF in temporal, parietal, frontal orbitofrontal cortex and temporal regions such as hippocampus, amygdala, and thalamus
Zhu et al. [49]	MRS	14 AD, 22 CN elderly subjects	Increased mI, mI/Cr and decreased NAA and NAA/Cr ratios were found in parietal areas for patients with AD. NAA/mI ratios were the best classifier for AD
Tumati et al. [50]	MRS	Meta-analysis of 29 studies	In the posterior cingulate, Cho/Cr ratios are increased, and NAA/mI ratios are decreased for AD patients. In the hippocampus, mI/Cr ratios are increased for AD patients

Changes in the neurovasculature system, namely in cerebral blood flow (CBF), can be detected by MR imaging using ASL (Table 2). Notable hypoperfusion is present in the posterior cingulate, precuneus, and, occipital, temporal, parietal cortical areas in AD and MCI, and in frontal and orbitofrontal cortex, and the hippocampus in AD. AD patients demonstrate greater CBF declines in cortex found in temporal, parietal, frontal, and orbitofrontal areas, in addition to the thalamus and middle temporal structures including the hippocampus and amygdala when compared to those with MCI [41–43]. Limited increases in CBF have been shown in the basal ganglia, amygdala, and hippocampus in MCI, and anterior cingulate in AD, which suggests compensatory mechanisms within the brain for cerebrovascular damage [43]. Regarding disease-related outcomes, regional hypoperfusion is associated with progression from MCI to AD, in addition to cognitive and functional deterioration [44]. Measures of perfusion on ASL also discriminate AD from VaD, DLB, and FTD. Differential patterns of CBF reduction were shown in frontal and temporal areas when comparing AD to VaD. Whilst demonstrating the highest degree of hypoperfusion throughout the brain, temporal regions are spared in DLB. In comparison, reduced temporal and frontal CBF is characteristic of AD and FTD, respectively [45, 46]. ASL utilizes magnetically labelled blood water as a tracer and individual differences in blood vessel properties could lead to variable transit times for its delivery. This might result in artificial changes in signal intensity, which a clinician might mistake as a disease-related abnormality in CBF. Another barrier to the employment of ASL in clinical practice is its low signal to noise ratio, which leads to reductions in image quality [47].

MRS assesses brain metabolite levels and its parameters are expressed as concentration or ratios to standardize values [48] (Table 2). When examining region-specific changes in AD, lower N-acetylaspartate (NAA) and NAA/Creatine(Cr) and higher myo-Inositol (mI) and mI/Cr ratios are found in parietal regions. Parietal NAA/mI ratios are also deemed a valid discriminator of AD [49]. In MCI, NAA/mI ratios are lowered and Choline(Cho)/Cr ratios are increased in the posterior cingulate gyrus, whereas mI/Cr ratios are increased in the hippocampus [50]. Clinically, decreased NAA markers are predictive of phenoconversion to dementia and cognitive dysfunction [51, 52]. NAA/Cr and NAA/mI ratios discriminate AD from VaD, and glutamate/Cr ratios differentiate DLB from AD. Metabolic ratios are substantially lower in AD patients compared to VaD, but higher in widespread brain regions relative to DLB [53, 54]. Whilst MRS is able to study molecular processes in the brain non-invasively without exposure to ionizing radiation, this technique is limited by its low sensitivity [55]. Resultant attenuated signal strength makes it difficult to recommend its use by clinicians for diagnostic purposes in AD and MCI.

## Functional imaging

Functional MRI generates dynamic representations of brain activity through bold oxygen level-dependent (BOLD) signal, which measures changes in blood flow and volume [56] (Table 3). On memory tasks, patients with AD show no or less activation of hippocampal and other medial temporal structures when compared to controls. Findings of increased brain activity during encoding in parietal and posterior cingulate areas indicate some degree of compensation by the brain in lieu of medial temporal dysfunction [57, 58]. Patients with MCI have demonstrated similar hippocampal deactivation to those with AD during recall [59], but with hyperactivation during encoding phases [60, 61], which might underline mechanistic compensation in prodromal stages. fMRI findings in AD extend to tasks of working memory, visuospatial ability, attention, semantic knowledge, and motor performance [62–66] and in MCI tasks of attention and working memory [62, 64, 67].

**Table 3:** Research studies examining region-specific patterns of neuropathology in AD and MCI using functional MRI

Study	Imaging modality	Sample	Main findings
Small et al. [57]	Task-based fMRI	4 AD, 12 subjects with isolated memory decline, 4 controls	Reduced activation in regions of the hippocampus was found during a facial recognition task for AD patients. A similar finding was observed for patients with isolated memory decline
Sperling et al. [58]	Task-based fMRI	7 AD, 10 young control subjects, 10 elderly control subjects	Reduced activation in hippocampal areas and increased activation in the parietal regions and the posterior cingulate were found during an encoding task for AD patients
Petrella et al. [59]	Task-based fMRI	13 AD, 34 aMCI, 28 healthy elderly control subjects	Decreased activation was found in middle temporal areas and increased activation was shown in posteromedial cortical regions for AD patients during an encoding task. Patients with MCI showed an intermediate but similar profile
Trivedi et al. [60]	Task-based fMRI	16 aMCI, 23 controls	Reduced activation was noted in frontal areas and increased activation was present in hippocampal areas for MCI patients during an encoding task. During recognition, this region-specific pattern of activation was reversed
Parra et al. [61]	Task-based fMRI	10 AD, 10 MCI, 10 controls	Comparing control subjects and MCI patients, decreased activation was found in the hippocampus and parahippocampus in AD patients during incidental encoding. Increased activation was found for MCI patients relative to control subjects
Yetkin et al. [62]	Task-based fMRI	11 AD, 10 MCI, 9 controls	Increased activation in frontal and temporal regions, fusiform gyrus, and anterior cingulate gyrus was displayed for AD and MCI patients during a working memory task. For selected areas, MCI patients showed greater activation than AD patients
Thiyagesh et al. [63]	Task-based fMRI	12 AD, 13 elderly control subjects	Declines in activation in parietal, parieto-occipital, and premotor cortical areas and increased activation of additional parietal structures was found in AD during an observational visuospatial task
Li et al. [64]	Task-based fMRI	10 AD, 9 MCI, 9 elderly control subjects	Reduced activation was found in prefrontal cortical areas for AD patients and increased activation in these same regions was found for MCI patients during a Stoop colour–word interference task
McGeown et al. [65]	Task-based fMRI	29 AD, 19 controls	No activation in parietal regions and decreased activation in prefrontal areas was found for AD patients during a semantic knowledge task
Vidoni et al. [66]	Task-based fMRI	9 AD, 10 controls	Reduced activation was found in the premotor and supplementary motor regions, and the cerebellum, whilst increased activation was evidenced in the primary motor cortices for AD patients during a motor task
Van Dam et al. [67]	Task-based fMRI	8 aMCI, 8 controls	Increased activation was shown in the tempero-parietal junction, angular gyrus, and precuneus, whereas attenuated activation was seen in prefrontal regions and the anterior cingulate for aMCI patients during an attentional (executive control, alerting and orienting) task
Greicius et al. [69]	Resting-State fMRI	15 AD, 18 controls	Reduced connectivity was shown between medial temporal structures and the posterior cingulate cortex for AD patients
Damoiseaux et al. [71]	Resting-State fMRI	Baseline: 21 AD, 18 controls Follow-up: 11 AD, 10 controls	Compared to control subjects at baseline, declines in connectivity were seen in the posterior DMN and increased activation was found for areas within the ventral and anterior DMN for AD patients. Compared to control subjects at follow-up, decreased connectivity between regions within the anterior, ventral, and posterior DMN in addition to sensorimotor network were shown for AD patients. Compared to control subjects, declines in activation over time were greater for AD patients
Yu et al. [72]	Resting-State fMRI	32 AD, 26 MCI, 58 controls	Increased connectivity between posterior cingulate and non-DMN regions but declines in activation between the posterior cingulate and areas within the DMN were found for AD patients. An opposite pattern of connectivity was shown for MCI patients
Das et al. [73]	Resting-State fMRI	17 aMCI, 31 controls	A greater degree of functional connectivity was shown within regions belonging to the medial temporal lobe, whereas declines in activity were seen between DMN and medial temporal structures for MCI patients
Zhou et al. [74]	Resting-State fMRI	35 AD, 27 MCI, 27 controls	Declines in functional connectivity within a range of regions within the thalamo-cortical network and thalamo-DMN were observed for AD patients. MCI patients showed similar but intermediate deteriorations
Li et al. [75]	Resting-State fMRI	15 AD, 16 healthy elderly control subjects	Declines in functional connectivity within a range of regions within the dorsal attention network but not the ventral attention network were found for AD patients
Zheng et al. [76]	Resting-State fMRI	32 AD, 38 controls	Disturbed functional connectivity was seen in several main networks including the DMN, visual network, and sensorimotor network in AD patients

Resting-state fMRI provides insight into functional connectivity among structures in intrinsic networks implicated in the AD spectrum (Table 3). One particular network of interest is the default mode network (DMN), where increased neural activity is shown at rest compared to task engagement. Brain structures implicated in the DMN include the posterior cingulate cortex (PCC), ventral anterior cingulate cortex, medial prefrontal cortex, inferior parietal cortex, dorsolateral prefrontal cortex, inferolateral temporal cortex, orbitofrontal cortex, and parahippocampal gyrus [68]. Abnormal coactivation at rest in AD was shown between medial temporal structures such as the hippocampus and entorhinal cortex and the posterior cingulate cortex (PCC) [69]. This evidences the significance of the MTL in the DMN and establishes altered connectivity in the DMN as an indicator for AD. Levels of PCC connectivity to other DMN structures is associated with neuropsychological impairment and declines in PCC-retrosplenial cortex connectivity is associated with lower Aβ levels in the CSF for AD patients [70].

There is a decrease in posterior and an increase in anterior and ventral DMN regions early in AD. 2–4 years later all regions show marked declines in connectivity [71]. This supports the notion that early mechanistic compensation occurs intrinsically within the DMN, but eventually global neurodegeneration occurs. This pattern of DMN dysfunction has been noted in MCI with limited increases in activation between DMN structures, indicative of prodromal compensatory mechanisms [72, 73]. Other large-scale brain networks that show disruption in AD include thalamo-cortical, dorsal attention, visual, and sensorimotor ones [74–76]. Whilst fMRI provides unique insight into pathophysiology, its use in the clinical routine is not supported [77]. This is due to primary limitations including a low signal or contrast to noise ratio and the questionable validity of BOLD signal as a measure of neuronal activity. Unexplained variability in this signal might result from hemodynamic factors that are not controlled for [78].

## Conclusions

AD is a devastating illness that leads to cognitive impairment and functional deterioration. MRI modalities have shown substantial utility in identifying biomarkers for AD and MCI pathology. These, in turn, can be used to improve diagnostic accuracy and develop novel molecular-based treatment interventions. Whilst only traditional structural modalities are recommended for diagnosis in clinical practice of MCI and AD, there is a need for further research to overcome methodological limitations of more advanced ones, which provide unique insight into disease-specific patterns of neuropathology. This should hopefully warrant their inclusion in diagnostic criteria for MCI and AD in the future.
